# Differentiation of Hamartomas and Malignant Lung Tumors in Single-Phased Dual-Energy Computed Tomography

**DOI:** 10.3390/tomography10020020

**Published:** 2024-02-11

**Authors:** Moritz T. Winkelmann, Sebastian Gassenmaier, Sven S. Walter, Christoph Artzner, Konstantin Nikolaou, Malte N. Bongers

**Affiliations:** 1 Department for Diagnostic and Interventional Radiology, University Hospital Tuebingen, 72076 Tuebingen, Germany; sebastian.gassenmaier@med.uni-tuebingen.de (S.G.); sven.walter@med.uni-tuebingen.de (S.S.W.); christoph.artzner@diak-stuttgart.de (C.A.); konstantin.nikolaou@med.uni-tuebingen.de (K.N.); malte.bongers@med.uni-tuebingen.de (M.N.B.); 2 Institute of Radiology: Diakonie Klinikum Stuttgart, 70174 Stuttgart, Germany

**Keywords:** dual-energy CT, hamartomas, malignant lung lesions, diagnostic accuracy

## Abstract

This study investigated the efficacy of single-phase dual-energy CT (DECT) in differentiating pulmonary hamartomas from malignant lung lesions using virtual non-contrast (VNC), iodine, and fat quantification. Forty-six patients with 47 pulmonary lesions (mean age: 65.2 ± 12.1 years; hamartomas-to-malignant lesions = 22:25; male: 67%) underwent portal venous DECT using histology, PET-CT and follow-up CTs as a reference. Quantitative parameters such as VNC, fat fraction, iodine density and CT mixed values were statistically analyzed. Significant differences were found in fat fractions (hamartomas: 48.9%; malignancies: 22.9%; *p* ≤ 0.0001) and VNC HU values (hamartomas: −20.5 HU; malignancies: 17.8 HU; *p* ≤ 0.0001), with hamartomas having higher fat content and lower VNC HU values than malignancies. CT mixed values also differed significantly (*p* ≤ 0.0001), but iodine density showed no significant differences. ROC analysis favored the fat fraction (AUC = 96.4%; sensitivity: 100%) over the VNC, CT mixed value and iodine density for differentiation. The study concludes that the DECT-based fat fraction is superior to the single-energy CT in differentiating between incidental pulmonary hamartomas and malignant lesions, while post-contrast iodine density is ineffective for differentiation.

## 1. Introduction

Incidental pulmonary nodules, which are defined as opacities with distinct and irregular margins of ≤3 cm in size and are surrounded by aerated lung parenchyma, are a common finding in thoracic computed tomography (CT) diagnostics [1].

In positron emission tomography (PET), unexpected malignant or premalignant lesions occur in 1.7% of 1750 patients [2]. Although no precise incidence figures were available for computed tomography, expected and unexpected malignant lesions of the lung are common, especially in the context of staging CT. In patients with a known tumor disease, multiple solitary pulmonary nodules are often compatible with lung metastasis. However, solitary pulmonary metastases can also mimic primary lung carcinoma. With imaging alone, it is thus sometimes difficult to determine whether it is a primary lung cancer or lung metastases [3].

The etiology of benign lung lesions is also diverse and includes benign infectious causes such as round pneumonia, septic emboli and granulomas, non-infectious causes such as amyloidosis, subpleural lymph nodes, rheumatoid nodules, granulomatosis with polyangiitis, or tumors such as hamartoma, carcinoid or neurofibroma [4]. 

In incidentally detected pulmonary nodules on conventional imaging, a small size and smooth, well-defined margins are indicative of benignity but are not diagnostic [5]. An irregular or tapering margin with distortion of the adjacent vessels may indicate malignancy, and there is considerable overlap between benign and malignant nodules in the internal characteristics of the nodules (e.g., cavitation, wall thickness, attenuation) [5].

Hamartomas are the most frequently detected benign lung lesions, and they usually are asymptomatic and do not transform into malignant lesions [6]. However, patients with pulmonary hamartomas may also suffer from malignant primary tumors that have the potential to develop pulmonary metastases, or malignant lung lesions may already coexist, which can complicate the differentiation of these lesions in the initial staging CT [6,7]. CT features indicating pulmonary hamartoma include intranodular fat and popcorn-like calcifications. However, fatty deposits in pulmonary hamartomas are detectable in 30–60% of cases on conventional CT images, and popcorn-like calcifications are only detectable in 10–30% of cases [8,9,10,11]. The application of single-energy CT (SECT) for early the differentiation between benign and malignant lung lesions is often based on morphologic features, which remains controversial [12,13].

In order to precisely differentiate between hamartomas and malignant pulmonary lesions, additional diagnostic procedures have been used so far, such as interval follow-up CT, PET-CT or lung biopsy [14]. An unambiguous diagnosis of hamartoma often requires enucleation of the tumor followed by histopathological analysis [15]. Diagnosis by fine needle biopsy cytology showed a specificity of 78% and a false-positive rate of 22% [16]. However, further medical examinations or interventions can increase costs, radiation doses and the risk of complications for patients.

Patients would benefit from faster and more cost-effective differentiation, such as single-phase dual-energy CT, for this reason, as the early identification of malignant nodules is essential to guarantee a better outcome for the patients [17]. In that regard, dual-energy CT (DECT) offers several clinical benefits over single-energy CT. In CT imaging, x-rays produce a spectrum of photon energies, with the highest energy corresponding to the kilovoltage of the tube measured with aluminum or copper absorbers [18,19,20]. Dual-energy CT (DECT) exploits this to improve the material contrast by comparing attenuation across different energies, with virtual monochromatic images (VMIs) at 65–70 keV providing data equivalent to 120 kVp scans at one energy [21]. DECT excels at differentiating materials of a similar density by using the CT figures of two energy levels for improved tissue characterization [20]. This technology, which has advanced medical imaging, captures low- and high-energy images simultaneously, thereby overcoming the ambiguity of voxel values in single-energy images that may not accurately represent tissue densities or contrast agent concentrations [22,23]. By merging images with different energies, DECT can reconstruct material-specific images, including virtual non-contrast (VNC) images, iodine quantification and fat fraction analysis [24]. The analysis can either be image-based for various post-processing applications or raw data-based for direct material dissection, with the choice of method depending on the hardware of the CT system [25]. Dual-energy technologies achieve this through methods such as fast switching, sequential scanning, dual-source systems or beam splitting at the source or detector [20].

We hypothesized that at portal venous CT, quantitative DECT analysis may help differentiate incidental pulmonary hamartomas from malignant lung lesions.

The purpose of this study was to evaluate the accuracy of single-phase dual-energy CT (DECT) with virtual non-contrast (VNC), iodine, and fat quantification to differentiate between pulmonary hamartomas and malignant lung lesions.

## 2. Methods and Materials

### 2.1. Patient Population

The local institutional review board of our university hospital approved this retrospective single-center study and waived the requirement for written informed consent (IRB code: 708/2019BO2). 

A computerized search of our patient database was performed by a board-certified radiologist to identify patients with incidental lung lesions who underwent DECT at our institution between November 2016 and January 2021. A total of 28 patients with hamartomas were identified. However, seven of these patients had to be excluded due to missing or insufficient reference standards. The final patient cohort with hamartomas consisted of twenty-one patients with twenty-two pulmonary hamartomas.

**Figure 1:**
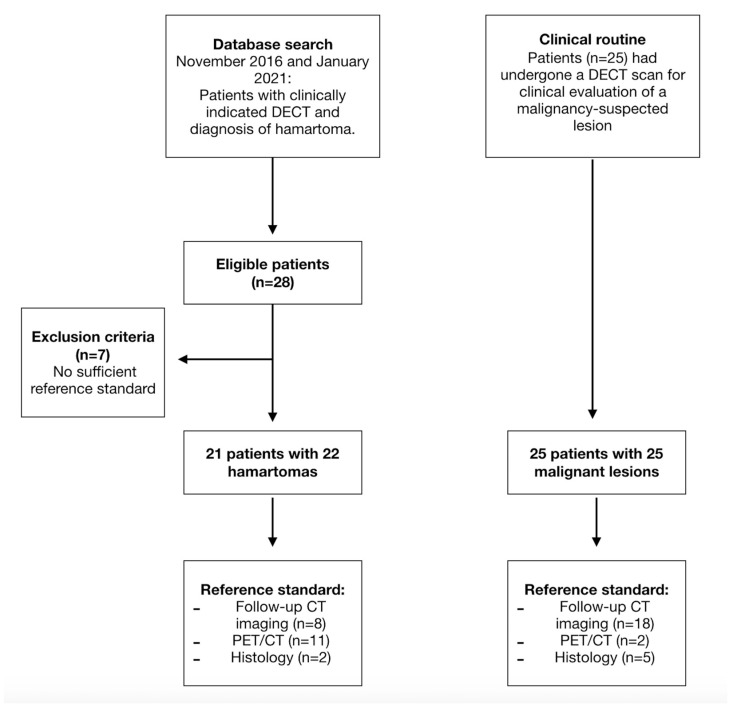
Flowchart of the two study arms (hamartoma and malignant lung lesions) and the corresponding number of patients included and excluded as well as the respective reference standards that led to the diagnosis.

During the same period, a high number of patients (*n* > 500) with malignancy-suspected lung lesions who had undergone a staging DECT examination were found in the database search. To obtain a comparable group to the patients with hamartomas and to avoid selection bias, only the initial 25 patients with a sufficient reference standard were included. In all 25 patients, the suspected diagnosis was confirmed by CT follow-up, PET-CT or biopsy (Figure 1). The final study cohort included 46 patients.

### 2.2. Acquisition Parameters

Each dual-energy CT scan was conducted using a third-generation dual-source CT system (SOMATOM Force, Siemens Healthineers, Erlangen, Germany). Imaging was performed in the portal venous phase, initiated 80 s after the administration of a body weight-adjusted contrast medium (0.5 mL/kg, Imeron 400, Bracco, Milan, Italy), delivered at a rate of 2.0 ± 0.5 mL/s using a dual-syringe injection system (Medrad, Bayer, Leverkusen, Germany) and followed by a 40 mL saline flush. Image acquisition utilized dynamic tube current modulation technology (CareDose4D, Siemens Healthineers, Erlangen, Germany) to optimize the dose during the scans. The dual-energy CT protocol included settings of 100 kV for tube A and tin-filtered 150 kV (Sn150 kV) for tube B, which were calibrated to reference tube current–time products of 190 mAs for tube A and 95 mAs for tube B. The system featured a collimation of 0.6 × 192 mm, a helical pitch of 0.6 and a gantry rotation duration of half a second. Reconstructions of the dual-energy series were generated on a specialized DECT workstation (syngo.via VB10B, Siemens Healthineers) in both the axial and coronal planes with thin 1.5 mm slices.

### 2.3. Dual-Energy Image Analysis

The images were analyzed using a custom workstation (syngo.via, version VB10B, Siemens Healthineers, Erlangen, Germany), which used an iodine subtraction technique (liver VNC in syngo.via, version VB10B, Siemens Healthineers, Erlangen, Germany) to simulate virtual non-contrast (VNC) in addition to quantifying iodine density and percentages of fat. The platform facilitates the spectral evaluation of different materials by performing an image-based examination across the spectrum of low- and high-energy kV peaks. It generates an iodine distribution map for each voxel using a three-material decomposition approach that assumes each voxel is composed of fat, soft tissue and iodine.

**Figure 2:**
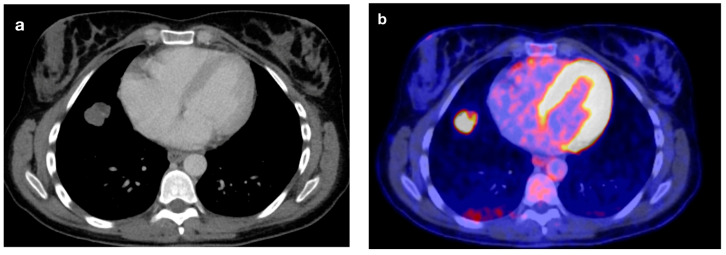
(**a**) A 31-year-old female patient with malignant melanoma and an initial suspicion of pulmonary hamartoma due to presumably macroscopically visible fat without a DECT follow-up. (**b**) The subsequent PET-CT a few weeks later shows an intensive FDG image. The lung lesion ultimately turned out to be the metastasis of a malignant melanoma.

**Figure 3:**
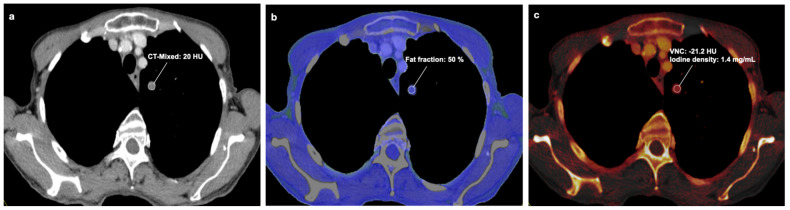
A 72-year-old patient who had undergone portal venous DECT and was found to have a 14 mm pulmonary nodule, which was subsequently diagnosed as a hamartoma. (**a**) Linearly blended DECT images are shown (equivalent to single-energy CT) with a CT mixed value of 20 HU. (**b**) The corresponding fat map in the same slice position shows a fat percentage of 50%. (**c**) The iodine mapping shows a ROI in the same slice position with a VNC of −21.2 HU and an iodine density of 1.4 mg/mL.

VNC, iodine concentration, fat content and CT mixed values (corresponding to conventional single-energy CT) were extracted for each lung lesion using DECT. These assessments were performed by a chest radiologist with four years of experience who did not know the final diagnosis of the lung lesions to ensure an unbiased measurement (Figure 2a,b and Figure 3).

Three different regions of interest (ROIs) were placed per lesion, and the mean value was calculated to avoid measurement inaccuracies. In hamartomas, the radiologist did not select ROIs exclusively within fatty regions, if visible, but rather equally distributed them throughout the lesion (Figure 3). ROIs in which calcifications were measured have been directly discarded.

### 2.4. Reference Standard

The histology reports, PET-CT examinations and follow-up CT thorax images were used as an internal reference by a radiologist (*blinded*) with 9 years of experience in thoracic imaging. The radiologist was blinded to the results of the DECT data analysis. Patients either underwent further CT imaging of the thorax after 6–12 months after the CT in which the measurement was taken (*n* = 26), histopathologic analysis (*n* = 7) or PET-CT (*n* = 13) to confirm the suspected diagnosis.

### 2.5. Statistical Analysis

Statistical analysis of the mean values obtained from the dual-energy CT (DECT) image data was performed using JMP 14 (SAS Institute Inc., Cary, NC, USA) and MedCalc Statistical Software version 18.1 (MedCalc Software bvba, Ostend, Belgium). Due to the skewed distribution of the data set, non-parametric methods, in particular that of the Wilcoxon test, were used in the analysis to allow for the comparison of mean virtual non-contrast (VNC), fat content, iodine density and CT mixed values—representing single-energy CT values—between the DECT images of pulmonary hamartomas and malignant lung lesions. Diagnostic efficacy was determined by calculating the receiver operating characteristic (ROC) curves, with the ideal threshold determined by the Youden index. Data are presented descriptively as medians, which include the 25th and 75th percentiles, and for normally distributed data as means with their standard deviations.

## 3. Results

### 3.1. General Analysis

The study population included 46 patients with a mean age of 65.2 ± 12.1 years. Twenty-one patients (mean age: 65.2 ± 12.1 years; female: *n* = 31; male: *n* = 15) were diagnosed with 22 hamartomas, and 25 patients had malignant lung lesions of which the most common primary tumors were melanoma, renal cell carcinoma, breast cancer and lung cancer. Most of the hamartomas were round in structure and only six of them had calcifications. Most malignant lesions were oval in configuration, and no calcifications were detectable. The diagnoses were confirmed by follow-up CT imaging, PET-CT or histology. Further patient and lesion characteristics are depicted in Table 1. 

### 3.2. DECT Image Analysis

The analysis revealed that there was a significant difference in the fat fraction values, with hamartomas having a median of 48.9% (interquartile range (IR): from 39.5% to 54.8%) and malignant lesions having a median of 22.9% (IR: from 12.8% to 27.1%) (*p* ≤0.0001). Virtual non-contrast (VNC) values on dual-energy computed tomography (DECT) also differed significantly, with hamartomas having a median of −20.5 Hounsfield units (HU) (IR: from −39.3 to −6.8 HU), while malignant lung lesions had a median of 17.8 HU (IR: from 3.8 to 29.4 HU), (*p* ≤0.0001).

**Figure 4:**
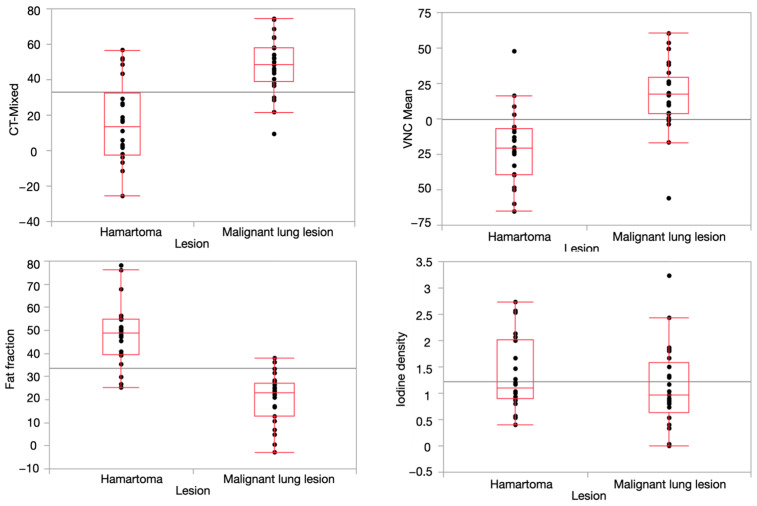
Boxplots show median and quantiles of CT mixed values, VNC, fat percentage and iodine density.

In addition, CT mixed scores were also able to differentiate between hamartomas with a median of 13.7 HU (IR: from −2.4 to 32.7 HU) and malignant lung lesions, which had a significantly higher median score of 48.3 HU (IR: from 39.1 to 57.9 HU) (*p*-value ≤ 0.0001). However, there were no statistically significant differences in the iodine density between hamartomas and malignant lesions, with the former having a median of 1.1 mg/mL (IR: from 0.9 to 2.0 mg/mL) and the latter having a median of 1.0 mg/mL (IR: from 0.6 to 1.6 mg/mL) (Figure 4).

### 3.3. Diagnostic Accuracy

In the ROC analysis, the fat fraction was shown to be the most accurate parameter with an AUC of 96.4%, significantly outperforming virtual non-contrast (VNC) images and CT mixed images, which had AUCs of 88% and 85.6%, respectively (*p* < 0.0001 for both comparisons). Iodine density analysis had the lowest AUC at 60.6%, with a *p*-value of 0.2.

Sensitivity and specificity for the fat fraction were particularly high, achieving a sensitivity of 100% and a specificity of 81.8%. The VNC and CT mix parameters had an equally high sensitivity and specificity of 88% and 81.8% for VNC and 88% and 77.3% for CT-mixed, thereby indicating high diagnostic performance (*p* < 0.0001 for both). In contrast, iodine density had a lower sensitivity of 44%, although it maintained a specificity of 77.3% (*p* = 0.2).

**Figure 5:**
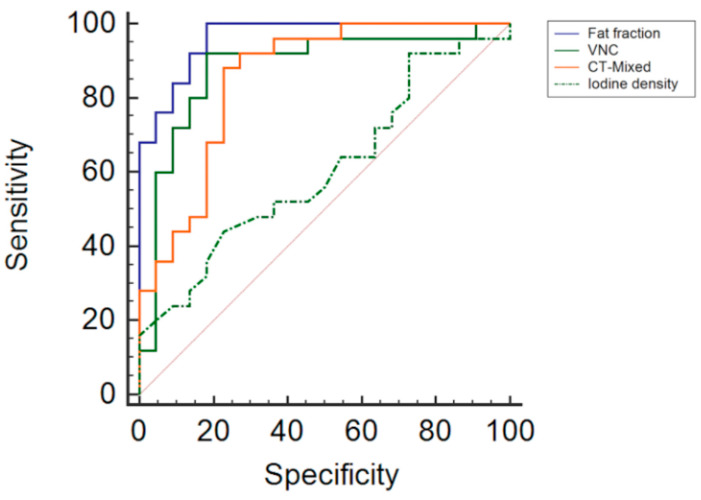
Comparison of ROC curves for fat fraction, VNC, CT mixed values and iodine density.

To further refine the clinical utility of these findings, optimal thresholds for the differentiation of lung lesions were established. A threshold of ≤38% was set for the fat fraction indicating benign lesions. The threshold value for the VNC was set at >−5.6 HU and for CT mixed at >29.2 HU, with these values being indicative of malignant disease. A value of ≤0.87 mg/dL was set as the threshold for iodine density (Figure 5; Table 2).

## 4. Discussion

The aim of this study was to evaluate the feasibility of DECT in distinguishing pulmonary hamartomas from malignant tumors of the lung by extracting VNC, iodine quantification and fat fraction analysis using single-phase portal venous DECT. DECT is a relatively new technology that has been used in several studies to differentiate between benign and malignant tissue in various organs [26,27,28]. One study prospectively examined 125 patients with 126 lung lesions and attempted to differentiate malignant from benign lesions using DECT parameters such as absorption in Hounsfield units, effective atomic number, iodine concentration and spectral CT curves. However, the study concluded that while the minimum effective atomic number and normalized mean effective atomic number were significant discriminators, normalization for the paravertebral muscle was not reliable due to poor reproducibility. It was suggested that a quantitative approach using raw measurements is simpler than one using logistic regression models [29]. Another study focused on the differentiation between malignant and benign necrotic lung lesions using dual-energy spectral CT. The study emphasized the use of dual-energy spectral CT with kVp switching, which allows for more quantitative parameters to be determined, improving the ability to differentiate between necrotic lesions [30]. However, the use of quantitative parameters such as VNC, fat fraction and iodine with dual-energy CT for the differentiation of malignant lung lesions and hamartomas has not yet been widely discussed in the literature.

By removing the iodine data, VNC can generate images that closely resemble actual non-contrast images, significantly reducing the radiation dose when detecting and assessing bleeding or delineating organ lesions [31,32,33,34]. Iodine quantification using DECT serves as an indirect measure of tissue perfusion and has shown promising results in the identification of lymph node metastases, the detection of the early stages of acute pancreatitis and the differentiation of intraperitoneal hematomas and the intestines [35,36,37,38]. 

By means of DECT-based fat quantification, on the one hand, it is possible to accurately determine, e.g., the liver fat content [39,40], but on the other hand, the relative tissue fat content can also represent additional information for the delimitation of differential diagnoses in different pathologies. It has been demonstrated that adrenal and hepatic lesions can be differentiated by DECT fat quantification [27,41]. 

The results of this study may have clinical implications. Contrast-enhanced DECT quantification of the fat fraction may help to accurately differentiate incidentally detected hamartomas from malignant pulmonary lesions. This may reduce the need for additional investigation. This study demonstrated a superior performance for fat fraction analysis in differentiating between hamartomas and malignant pulmonary lesions compared with the assessment of conventional contrast-enhanced images (CT mixed). VNC and CT mixed images are also strong discriminators between hamartomas and malignant pulmonary lesions but with a lower sensitivity compared with the fat fraction. No significant difference in the iodine density was observed between hamartomas and malignant lung lesions, suggesting that contrast uptake occurs in hamartomas, possibly mainly in the septa corresponding to loose connective tissue rather than the cartilaginous tissue [11]. This implies that contrast enhancement needs to be reconsidered as an indicator of malignancy in pulmonary lesions [42].

A case study already suggested that the DECT-based detection of intranodular fat could help in the diagnosis of pulmonary hamartomas [15]. Detectable intralesional adipose tissue on conventional CT is often used to aid in the diagnosis of pulmonary hamartoma [8]. However, only 30–60% of pulmonary hamartomas have fatty deposits that are detectable on conventional CT [8,11,12,43]. Fat fraction analysis using DECT might be useful for the detection of intralesional fat in patients in whom no macroscopic fat is detectable in single-energy CT images. DECT analysis for a tissue feature assessment is based on the unique CT attenuation characteristics of different materials at different x-ray energy levels. Adipose tissue has been reported to possess unique CT attenuation changes, and detecting a decrease in density from higher to lower kV images may be indicative of intralesional fat [44]. 

A potential explanation for the inferior performance of virtual non-contrast (VNC) values compared with that of fat fractions in distinguishing between hamartomas and malignancies may lie in the VNC’s vulnerability to residual iodine content following contrast enhancement. Evidence from multiple studies indicates that VNC images acquired during different vascular phases—specifically in the arterial and venous phase—exhibit notably disparate CT values. This variability could compromise the diagnostic reliability of VNC HU values in the post-contrast phase, thereby affecting their ability to accurately differentiate between these lesions [45,46]

We acknowledge that our study has limitations. A retrospective study design was utilized, and the study cohort was small. Because of potential case selection bias, diagnostic performance in this retrospective cohort should be considered with caution. The results of this study are intended to help differentiate hamartomas from malignant lung lesions in portal venous DECT. DECT fat quantification is not a substitute for other procedures for definitive diagnosis of lung tumors, such as a biopsy. Therefore, larger prospective studies should be designed to confirm the results of this study.

## 5. Conclusions

The DECT-based fat fraction of single-phased CT allows for the improved differentiation between incidental pulmonary hamartomas and malignant lung lesions compared with single-energy CT. Iodine density after contrast administration has a poor potential to discriminate between benign and malignant lung lesions.

## Figures and Tables

**Table 1:** Patient and lesion characteristics.

Variables	/Mean ± SD
Age (years)	65.2 ± 12.1
Male patients (n)	15
Female patients (n)	31
Hamartomas (n)	22
Mean lesion size (mm)	13.7 ± 7.3
Lesion characteristics	
- round	13
- oval	5
- lobulated	4
- calcifications	6
- macroscopic fat	9
Malignant lung lesions (n)	25
Mean lesion size (mm)	16.3 ± 7.5
Lesion characteristics:	
- round	8
- oval	11
- lobulated	6
- calcifications	0
Tumor entitiy:	
Melanoma (n)	4
Renal cell carcinoma (n)	4
Breast cancer (n)	3
Lung cancer (n)	3
Colorectal cancer (n)	2
Oropharyngeal cancer (n)	2
Prostate cancer (n)	1
Rhabdomyosarcoma (n)	1
Ewing-like sarcoma (n)	1
Soft-tissue sarcoma (n)	1
Clear cell sarcoma (n)	1
Gallbladder carcinoma (n)	1
Appendiceal carcinoma (n)	1

**Table 2:** Diagnostic performance in DECT image analysis.

	Fat Fraction	VNC	CT Mixed	Iodine Density
Area under the curve (AUC)	96.4%	88%	85.6%	60.6%
Standard error (AUC)	0.02	0.06	0.06	0.08
95% confidence intervalt	0.86–0.99	0.75–0.96	0.72–0.94	0.45–0.75
Optimal threshold	≤38%	>−5.6 HU	>29.2 HU	≤0.87 mg/dL
Sensitivity	100%	92%	88%	44%
Specificity	81.8%	81.8%	77%	77.3%

Of note: VNC, virtual non-contrast imaging.

## Data Availability

A data source can be provided upon request.

## Abbreviations

AUC	Area under the curve
CT	Computed tomography
DECT	Dual-energy computed tomography
FDG	Fluorodeoxyglucose
kV	Kilovolt
PET-CT	Positron emission tomography computed tomography
ROI	Region of interest
ROC	Receiver operating characteristic
SECT	Single-energy computed tomography
VNC	Virtual non-contrast
